# Sampling the conformation of protein surface residues for flexible protein docking

**DOI:** 10.1186/1471-2105-11-575

**Published:** 2010-11-23

**Authors:** Patricia Francis-Lyon, Shengyin Gu, Joel Hass, Nina Amenta, Patrice Koehl

**Affiliations:** 1 Department of Computer Science, University of California, Davis, CA 95616, USA; 2 Department of Mathematics, University of California, Davis, CA 95616, USA; 3 Department of Computer Science and Genome Center, University of California, Davis, CA 95616, USA

## Abstract

**Background:**

The problem of determining the physical conformation of a protein dimer, given the structures of the two interacting proteins in their unbound state, is a difficult one. The location of the docking interface is determined largely by geometric complementarity, but finding complementary geometry is complicated by the flexibility of the backbone and side-chains of both proteins. We seek to generate candidates for docking that approximate the bound state well, even in cases where there is backbone and/or side-chain difference from unbound to bound states.

**Results:**

We divide the surfaces of each protein into local patches and describe the effect of side-chain flexibility on each patch by sampling the space of conformations of its side-chains. Likely positions of individual side-chains are given by a rotamer library; this library is used to derive a sample of possible mutual conformations within the patch. We enforce broad coverage of torsion space. We control the size of the sample by using energy criteria to eliminate unlikely configurations, and by clustering similar configurations, resulting in 50 candidates for a patch, a manageable number for docking.

**Conclusions:**

Using a database of protein dimers for which the bound and unbound structures of the monomers are known, we show that from the unbound patch we are able to generate candidates for docking that approximate the bound structure. In patches where backbone change is small (within 1 Å RMSD of bound), we are able to account for flexibility and generate candidates that are good approximations of the bound state (82% are within 1 Å and 98% are within 1.4 Å RMSD of the bound conformation). We also find that even in cases of moderate backbone flexibility our candidates are able to capture some of the overall shape change. Overall, in 650 of 700 test patches we produce a candidate that is either within 1 Å RMSD of the bound conformation or is closer to the bound state than the unbound is.

## Background

This paper concerns one aspect of the problem of predicting the interface region of two docking proteins, namely the flexibility of side-chains. We consider sampling the possible side-chain conformations to improve the match of local shape complementarity. The main challenge we face is reducing the exponential complexity of the conformation space to a reasonably sized sample.

Proteins are at the heart of all biological processes, and their functions are largely determined by their geometric structures. The number of biomolecular protein complexes is expected to be far more than the number of individual proteins in a given proteome; in addition, their structures are more difficult to obtain through NMR and X-ray crystallographic studies. So the difficult problem of docking, that is, the computational prediction of a protein complex from the structures of its constituent proteins, is an important focus of current research. The constituent structures may be determined experimentally or may be computed by a protein structure prediction algorithm.

Docking is a difficult problem and it has received much attention [1-6]. In the docked configuration, two proteins demonstrate excellent shape complementarity, with the molecular surfaces matching each other closely over an interface region which is several square Å in area. This excellent fit is difficult to find, however, since proteins are not rigid. The protein backbone may undergo significant changes when docking. Even in the absence of large backbone motions, the conformations of the side-chains in the docked complex may differ greatly from their conformations in the unbound proteins; for instance, a side-chain may need to rotate to allow space for the docking partner. Although side-chain conformation changes are typically small compared to the larger backbone motions, they can be crucial in forming the tight fit between docked proteins. In this work, we consider the problem of producing candidate docked side-chain conformations, given the structure of the unbound protein. We focus solely on the flexibility of side-chains and as a first approximation we ignore backbone flexibility.

Docking methods currently incorporate conformational changes upon binding in different ways. The most common approach adopts an implicit "soft" surface representation that allows some degree of penetration of rigid proteins [3,7-10]. The rigid protein is rotated and translated with respect to its partner. Each configuration is evaluated by a scoring function which usually measures shape complementarity as well as chemical and physical information. The scoring function is adjusted to accommodate some steric clash at the surface and therefore accounts implicitly for flexibility. These algorithms are usually implemented as low-resolution methods due to the computational complexity of sampling the six-dimensional space of rigid motions. But as computers become more powerful the resolution of these methods cannot be substantially improved, since the "soft" surface representation is required to mask the difference between docked and undocked conformations.

Soft rigid-body docking methods are generally followed by a refinement stage in which an atomic-level energy minimization is carried out to allow for explicit flexible protein-protein docking and then by an all-atom rescoring scheme. The HADDOCK [11] algorithm, for example, implements this refinement in a three-stage process: rigid body energy minimization followed by two refinement steps: semirigid simulated annealing in torsion angle space and refinement in Cartesian space with explicit solvent. Backbone and side-chains flexibility is allowed during refinement. RDOCK [12] is an energy minimization process on side-chains that refines ZDOCK's [7] initial rigid-body docking candidates. Molecular dynamics simulations (MD) have also been used to refine both side-chain and backbone conformations [13,14].

Alternative approaches to the soft rigid body docking procedures described above account for flexibility by considering multiple conformations of the proteins involved in the complex. These conformations correspond to alternative models (such as those derived from NMR studies), models constructed from point mutation studies, or models derived from dynamics simulations. This has been referred to as "cross docking" or "ensemble docking" [1] and has been implemented in protein-ligand docking programs [15,16].

Side-chain flexibility needs to be accounted for in the docking process. Cherfils et al. [17-19] introduced docking methods that represent side-chains with a crude low-resolution model to account for flexibility. Jackson et al. [20] include side-chain flexibility in a two-step process for interface refinement that is iterated until convergence. In the first step, the self-consistent mean field (SCMF) algorithm is used to find the optimal side-chain conformation of the protein from its rotamer states, taking solvation into account. In the second step rigid-body minimization of the intermolecular interaction energy is performed on the interface region only, while the larger molecule is held stationary. In the software ATTRACT, Zacharias [21] incorporated flexibility by using a rotamer library having up to three pseudo atoms for each amino acid [22]. In RosettaDock, Wang et al. [23] begin with a discrete rotamer library supplemented with side-chain conformations taken from the unbound structure, and then perform continuous optimization of side-chains in the vicinity of the rotamers.

Our approach to the docking problem is related to the ensemble docking model. We propose a strategy that includes three steps: (i) generate ensembles of conformations for multiple patches that span the surfaces of the two proteins considered, (ii) develop a fast method for generating candidate docked conformations of the two proteins based on these ensembles, and (iii), develop an energy function that ranks the candidate conformations such that the actual native docked structures can be identified. In step (i), we acknowledge the fact that proteins may change shape upon binding; this is implicitly captured by the ensemble of conformations, with the hope that this ensemble includes a conformation that is close to the conformation found in the bound protein. In step (ii), the major difficulty is to overcome the combinatorial explosion that arises from accounting for multiple patches, each represented with an ensemble of conformations. Fortunately, solutions are available. Firstly, it is possible to test the complementarity of two patches by mapping the two patches onto a feature space and by directly comparing their representations in this space. Secondly, the combinatorial explosion of handling multiple patches can be significantly reduced using a geometric hashing technique [24]. Finally, in step (iii) we anticipate that both geometry and energetics need to be incorporated into the scoring function for this function to be selective.

In this paper we focus on step (i) of the procedure outlined above. We explore the question of how well we can sample side-chain conformations so as to approximate the bound conformation of the protein. We leave the backbone conformation fixed as observed in the unbound state. Even with this fixed backbone, sampling all possible states of the side-chains is of course not computationally feasible. For a medium-sized protein of 250 residues, if each residue were allowed an average of 5 sampled states, the number of possible side-chain configurations would be on the order of 10 raised to the 175th power. In order to reduce this exponential complexity, we decompose the protein surface into a set of overlapping small patches; only the interactions between side-chains within each patch are considered.

Our main contribution is a procedure that produces a small sample of the space of side-chain configurations within a patch. We test our procedure using the docking benchmark of Hwang et al. [25]. This data set includes pairs of proteins for which the geometry of the bound configuration, as well as the unbound, is known for each protein. For each patch, we compute 50 sample configurations (see Methods) and we compare the experimental conformations we produce to the bound conformation. Our results are given in the Results and Discussion section. We find that in most cases our method produces a docking candidate that is within 1 Å RMSD of the bound configuration.

An ensemble of 50 configurations for each surface patch produces a very large space within which we need to search for shape complementarities. If, however, we can require better shape complementarity, at higher resolution, in the first phase of docking, we may be able to reduce rather than increase the number of false positives which must be considered in later stages.

## Methods

### Outline

**Figure 1:**
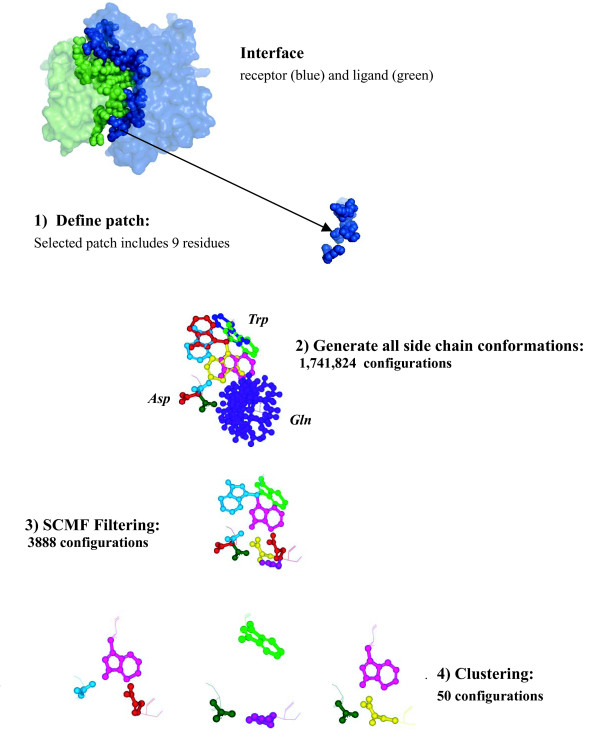
**Generating a sample of conformations for a protein surface patch**. We illustrate our method on the complex between the human MHC class I glycoprotein HLA-A2 and the T-cell co-receptor CD8, whose structure is available in the PDB [37] under the acronym 1AKJ: the interface is the opaque region where the receptor (HLA-A2: blue) and ligand (CD8: green) come into close contact. 1) The receptor is divided into patches; we select one that contains nine residues. 2) To allow for side chain flexibility, we generate different side chain conformations for each of the nine residues in the selected patch; as an illustration, we show 6 rotamers of a tryptophan (top), 3 rotamers of an aspartic acid, and 28 rotamers of a glutamine (purple). This results in 1,741,824 possible conformations for the patch. 3) These conformations are filtered according to an energy function using a self consistent mean field approach, 4) then clustered by k-means, reducing the number of docking candidates to 50.

Our overall goal is to represent a protein with an ensemble of conformations, which we refer to as a *sample*, in which side-chain flexibility is accounted for by sampling their possible conformations so as to include a good approximation to the bound state. The main challenge we face is to reduce the huge number of possible side-chain conformations so that we end up with a sample of reasonable size. To address this challenge, we employ a series of strategies that are schematically described in Figure 1.

Our first observation is that it is not necessary to represent conformations of the complete molecule, or even the complete molecular surface; the protein-protein interface region is usually a small part of the surface, so it suffices to represent conformations within local surface regions. We define small patches on the surface, consisting of overlapping sets of eight or nine residues. In an actual docking experiment, the set of patches would be chosen to cover the entire molecular surface. In this study, however, we only consider patches that actually intersect the interface region, so that we can compare our synthetically sampled conformations to the side-chain conformations in the docked (bound) complex.

To account for flexibility, we allow for different positions of the side-chains of the residues included in the patch, while leaving the backbone fixed. As in other methods for predicting the conformations of side-chains, we consider a discrete version of their conformational space, with each discrete conformation referred to as a rotamer [26]. These rotamers correspond to optimal, i.e. low energy states; they are usually grouped into libraries that have been compiled by performing statistical studies on known protein structures. An exhaustive enumeration of all possible rotamer combinations for the residues contained in a patch still leads to a very large number of conformations: in the example shown in Figure 1 the patch contains one tryptophan with 6 rotamers, two aspartic acids, each with 3 rotamers, one glutamine with 28 rotamers, two lysines, each with 8 rotamers, one arginine with 6 rotamers, one serine with 3 rotamers, and one alanine, leading to a total of:

6 × 3 × 3 × 28 × 8 × 8 × 6 × 3 = 1,741,824 possible conformations.

Taking just the lowest-energy conformations in this set would not provide a good sampling of the conformational space accessible by the patch. Indeed, conformations with similar low energies usually represent small variations on essentially the same local minimum. To provide a broader sampling, we subdivide the conformational space of each residue into thirds using its three common χ_1 _torsion angles (typically 60°, 180° and -60°). Our approach is to sample every possible χ_1 _configuration of the patch residues. With 9 residues to a patch, each having up to three χ_1 _angle possibilities, the number of χ_1 _configurations is up to 3^9^, ≈ 20,000. Instead of taking the lowest energy samples from among all exhaustive conformations, we select the lowest energy conformation of each of the approximately 20,000 χ_1 _configurations as follows. With the χ_1 _configurations fixed, we select the other torsion angles within the patch (χ_2 _and χ_3_) by energy minimization using the self consistent mean field (SCMF) algorithm, described below. The energy function we use includes both Van der Waals and Coulomb energies. Our final step is to use k-means clustering, based upon RMSD, to further reduce the set of about 20,000 configurations to a set of 50 clusters. One representative, that with the lowest energy, is selected from each of the 50 clusters, and the unbound configuration is added to form the final sample of docking candidates for the patch.

### Implementation

#### Data Set

We use in our experiments the protein-protein docking benchmark (version 3.0) developed by Hwang et al. [25]. This benchmark includes 124 "cases" in total. In general, a "case" represents a traditional unbound docking problem, including the unbound structures of the receptor and ligand proteins as well as the structure of the complex between these two proteins: 105 cases fall in this category. The other 19 cases are specialized problems representing antibody-antigen complexes for which only the bound structure of the antibody is known. The difficulty level of any case in the benchmark is measured by the degree of conformational changes that occur in and around the interface region upon binding, as measured by the interface C*_α_*-RMSD (I-RMSD) and by the fraction of non-native residue contacts (f*_non - nat_*). A case is deemed "rigid-body" and therefore "easy" if its I-RMSD is lower than 1.5 Å and its f*_non - nat _*is lower than 0.4. At the other end of the spectrum, a case is deemed "difficult" if its I-RMSD is greater than 2.2 Å. All the other remaining cases are referred to as "medium". Among the 124 cases, there are 88 rigid cases, 19 medium cases and 17 difficult cases.

#### Selecting patches

We test our conformation sampling method only in the interface regions of the two proteins of every test case, since only in the interface regions is it important to be able to approximate the bound conformation of the side-chains.

The first step is to define the interface regions. We designate a residue on a bound protein an interface residue if at least one of its atoms is within 6 Å of any atom of the complementary protein. This gives us a list of interface residues.

Our next step is to define a set of surface patches covering the interface regions. Patches are defined independently of each other on the two proteins. The objective is for the patches to be distributed evenly and with good overlap. Since the average size of an amino acid is about 8 atoms (excluding hydrogen) and we want each patch to contain eight or nine residues, we place a patch center at every 64*^th ^*position in the list of atoms in the interface region of one protein. Then we draw a sphere around each patch center. The radius of each sphere is adjusted until the patches contain either eight or nine effective residues of the interface; these residues define a patch. An effective residue is a residue that is neither an ALA nor a GLY (we separate these two types of amino acids from the other on the basis that their side-chains are not flexible, notwithstanding hydrogens). After this process, if there are any residues in the interface that have not been included in at least one patch, we set additional patch centers at their C*_α _*and repeat the sphere adjusting process to create new patches that cover the gaps.

The interface regions are defined according to the bound structures of the proteins. Mapping the bound patch residues to the corresponding residues in the unbound structure is not entirely straightforward, as there exist cases with (different) missing residues in the X-ray structures of the unbound and bound states of the protein. To circumvent this problem, we clean the data set using the following procedure on each case:

• We align the two sequences of the same protein extracted from the ATOM record of the PDB files for its bound and unbound conformation. If there are any missing or additional residues for the interface region in the unbound structure, the case is rejected.

• We "fix" any residues that are incomplete in the interface region for both the bound and unbound structure. By "fixing", we mean that if a residue is missing a side-chain atom, it is truncated to an ALA or a GLY (depending on whether the C*_α _*atom is present or not). We reject the case if any residue is missing a backbone atom.

• We select patches on the bound interface region using the procedure described above. Each patch contains eight or nine effective residues.

• Finally, we "fix" the non-interface region: (i) we remove all residues whose backbone is incomplete, or who are present in one of the two conformations (bound or unbound) but not in the other, and (ii) we truncate residues with missing side-chain atoms to ALA or GLY, consistently in the bound and unbound structure. If more than 5% of the residues are removed or truncated in this process, the case is rejected.

This stringent data cleaning led to many test cases being rejected: only 56 cases remained, including 10 antigen-bound antibody cases: they are listed in Table 1. From these, 700 patches were created. The number of patches per protein complex depends on the size of the two constituent proteins and on the shape of the interface region. We found an average of 12.5 patches per protein complex (i.e sum of the patches on the ligand and receptor proteins), with a minimum of 5 patches and a maximum of 22 patches. The size of these patches varies between 15 Å and 30 Å (where size is the maximal distance between any two atoms in the patch), with the smallest patch size at 14.55 Å and the maximum at 43.05 Å (average 20.75 Å).

**Table 1:** The different docking test cases included in our experiments

Complex PDB ID				
1ACB	1AHW	1AK4	1AKJ	1AY7
1B6C	1BJ1	1BKD	1BUH	1BVK
1BVN	1CGI	1D6R	1DFJ	1DQJ
1E6E	1E6J	1EAW	1EER	1EWY
1FC2	1FSK	1GHQ	1I9R	1IBR
1IQD	1KAC	1KTZ	1KXQ	1M10
1MAH	1ML0	1MLC	1NCA	1NSN
1QFW	1R0R	1S1Q	1SBB	1TMQ
1UDI	1VFB	1WEJ	1WQ1	1Y64
2AJF	2B42	2FD6	2I25	2JEL
2MTA	2QFW^a^	2SIC	2UUY	2VIS
7CEI				

^a ^Note that the name 2QFW does not correspond to the actual PDB file with this ID. the PDB file 1QFW corresponds to a ternary complex between human chorionic gonadotropin and two Fv fragments; it has been artificially separated into two binary complexes in the docking database of Hwang et al [25], with one complex stored as 1QFW and the other one stored as 2QFW

#### Rotamer library

As described in the overview, we allow side chain flexibility by having our docking candidates take on a sample of low-energy combinations of different torsion angles for each amino acid type. To reduce the size of the search space, we use only those torsion angles contained in a rotamer library [26]. Our rotamer library is a modified version of the averaged library compiled by Tuffery et al. [27], which has been corrected for duplicate rotamers. The backbone-independent rotamer library of Dunbrack and Cohen [28] was used for leucine residues. The missing dihedral angles in the Tuffery library χ_4 _for arginine and lysine were defined as 180 and χ_5 _for arginine as 0. To alleviate the effects of this approximation, we do not include the atoms defined by these dihedral angles in the energy calculations. Because there are many docked residues that stay close to their undocked conformations, we supplement our rotamer choices with the unbound conformation if there is no rotamer from the library that is within 0.6 Å RMSD of it.

**Figure 2:**
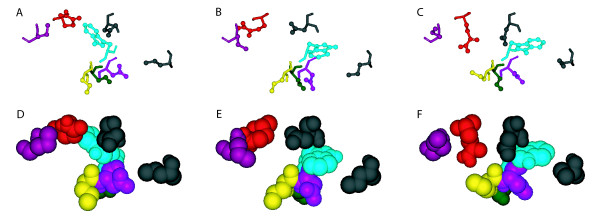
**Configurations of a patch**. Three configurations of the 1AKJ receptor patch in space filling mode (D, E, F) and with side chains viewed as ball and stick (A, B, C). The different configurations are seen easily in the top panel, and their impact on the shape of the patch is seen in the bottom. For example, the different rotamers of tryptophan (cyan) can be seen to affect the shapes of the 3 configurations of the patch.

The side chains are built upon the native backbone of the unbound protein using the torsion angles from the rotamer library and standard bond lengths and bond angles from the Charmm 19 force field. Figure 2 shows three different side-chain configurations for a patch from the 1AKJ receptor.

#### Energy function

We use a simplified physics-based energy function *E *that takes into account non-bonded interactions. It includes a Coulomb term and a Lennard-Jones term, which accounts for Van der Waals attractions and repulsions:

(1) $$E=\sum_{\left(i,j\right)}\min\left(\epsilon_{ij}\left(\frac{A_{ij}}{r_{ij}^{12}}-\frac{B_{ij}}{r_{ij}^{6}}\right),2\right)+\sum_{\left(i,j\right)}\frac{q_{i}q_{j}}{4\pi \epsilon_{0}\epsilon_{r}r_{ij}}$$

In this equation, *r_ij _*is the distance between atoms *i *and *j *that has been capped below at 2 Å, to circumvent problems that may come from using discrete rotamers (such as an atom being shifted from a favorable position to a new position leading to a severe steric clash). For the same reason, we use an upper cutoff value for Van der Waals energies of 2 Kcal/mol (introduced with the *min *function). Both summations run over all atoms *i *in the patch considered and all atoms *j *in the protein that contains this patch. The interaction is computed only if the two residues to which *i *and *j *belong have their C*_α _*atoms less than 20 Å apart. In addition, if *j *belongs to a residue that is accessible (i.e. whose accessible surface area is greater than 50% of its total accessible surface area), the interaction is scaled by a factor 1/2. Finally, the parameters *A_i,j_*, *B_i,j _*, ϵ_*i,j *_and the charges are taken from the CHARMM19 force field [29].

In addition, if the patch includes two cysteines, we add an additional geometric energy term that checks for disulfide bridge formation [30].

#### SCMF Filtering

The energy defined above is used in conjunction with mean-field theory to select low-energy conformations from the list of possible rotamer settings. Mean-field theory (MFT) is a way of addressing the combinatorial issues of a many-bodied system. In MFT, interactions with any one body are expressed as an average of interactions over an ensemble of states. When an exhaustive search of a space is intractable, and minimization occurs within the context of many local minima, MFT is an approximation that enhances the sampling protocol of the minimization algorithm. In its most general form, the MFT approximation can be applied to the average (denoted <*X *>) of any physical quantity *X*. <*X *> is expressed as the sum of the values of *X *for its subsystems weighted by their probabilities.

We employ the self-consistent mean field (SCMF) algorithm [30], an application of MFT to side-chain prediction. Consider a protein of *N *residues, with the *i^th ^*residue having *R_i _*rotamers. Assume that we are given a probability distribution on the rotamers. We replace the *i^th ^*side-chain with an ensemble containing all of the possible rotamer conformations, all attached to the original backbone at the C*_α _*atom. Each rotamer is weighted by its probability. All residues now interact with the entire rotamer ensemble.

To sum up the details (provided by Koehl and Delarue [30]) the effective energy of the multi-copy protein is given as:

(2) $$<E>=\sum_{i=1}^{N}\sum_{r_{i}=1}^{R_{i}}p_{ir_{i}}<E_{i}{>}_{r_{i}}$$

where *< E >_, _i*, *N*, *r_i _*and *R_i _*are as described above, $p_{ir_{i}}$ is the probability that the *i^th ^*residue is in the $r_{i}^{th}$ rotamer conformation, and $<E_{i}{>}_{r_{i}}$ is the effective energy felt by the *i^th ^*residue in its $r_{i}^{th}$ rotamer conformation, which is given by:

(3) $$<E_{i}{>}_{r_{i}}=E_{r_{i}}{}_{BB}+E_{r_{i}}+\frac{1}{2}\sum_{j=1}^{N}\sum_{r_{j}=1}^{R_{j}}p_{jr_{j}}E_{r_{i}r_{j}}$$

Here $E_{r_{i}BB}$ is the energy of interaction of the backbone with i's $r_{i}^{th}$ rotamer and $E_{r_{i}}$ is this rotamer's self energy. $E_{r_{i}r_{j}}$ is the energy of interaction of residue i's $r_{i}^{th}$ rotamer with residue j's $r_{j}^{th}$ rotamer.

We then define the effective free energy of the multi-copy system as:

(4) $$ℱ=<E>+\sum_{j=1}^{N}\sum_{r_{j}=1}^{R_{j}}p_{jr_{j}}\log\left(p_{jr_{j}}\right)$$

In this formulation, the problem of finding the global minimum of the energy landscape in the original conformational space is replaced by the problem of finding the minimum of the effective free energy given by equation 4 in a new conformational space where the degrees of freedom, i.e. the unconstrained values, are the probabilities. The energy function in this space is better behaved, with far fewer local minima [31]. The SCMF algorithm predicts protein side-chain configuration by choosing the highest probability rotamer for each residue.

SCMF optimization typically seeks only a single minimal configuration for the protein. As described in the Outline, we instead use SCMF as a filter to reduce our ensemble from an exhaustive sampling of all configurations of rotamers to a sampling covering the regions of χ_1 _space. Our approach is to sample each residue in turn in all its three χ_1 _angles of 60°, 180° and -60°. For each combination of χ_1 _angles of patch residues we create a multi-copy protein and have SCMF select the minimum energy configuration of the χ_2 _and χ_3 _torsion angles.

We do have two exceptions of this χ_1 _space discretization. Firstly, we retain exhaustive sampling of histidine as this residue is difficult to model given the ambiguity of its protonation state. We achieve better results by extensively sampling all of the histidine rotamers, each of which being protonated in the delta position. Secondly, we do not sample each of the 3 χ_1 _angles of serine and cysteine; rather, we allow the SCMF algorithm to select one unique conformation from their three rotamers.

#### Clustering

The SCMF filtering procedure significantly reduces the size of the sample of conformations for any given patch, to the roughly 20,000 conformations determined by the choices of χ_1 _angles. To reduce this size to something that would be reasonable to use in docking, we apply a clustering algorithm.

We use *k*-means clustering, with the distance between two conformations measured as the root mean square distance (RMSD) computed over all the side-chain atoms (we do not include the backbone atoms as they remain at the same position in all conformations). We selected *k *= 50 clusters as a good balance of small ensemble size and quality. We then select one representative for each cluster, chosen to be the conformation with the minimum energy, where the energy is given by equation 1.

#### Running time

Computing time is a key parameter for our procedure as it defines what are reasonable values for some of the parameters we use. Our procedure includes three steps, namely definition of the patches on the surface of the protein, generation and filtering of the conformations for each patch, and finally clustering of the resulting conformation to generate the ensembles of each patch. While the first step is fast, the second and third steps are controlled by the size of the patches, i.e. by the number of effective residues with flexible side-chains they contain. Patches with too many effective residues would lead to a combinatorial explosion in the process of exploring their conformational space. We have found that sizes of up to 9 residues are computationally tractable. The slowest step in our procedure is the clustering of the SCMF-filtered conformations. With patch size of up to 9 effective residues, we find that the average computing time for clustering is approximately 5 minutes per patch. This was computed on a desktop computer with an Intel Core 2 Quad CPU of 2.40 GHz, using one core per patch.

## Results and Discussion

We first reiterate our overall goal: to represent a protein patch with an ensemble of conformations that account implicitly for side-chain flexibility. This ensemble, or sample, must satisfy two seemingly contradicting criteria: it must cover as much as possible the conformational space accessible to the patch, so as to optimize the probability that it includes a conformation close to the one present in the bound complex, and it must be as small as possible so as to remain manageable when used in docking experiments. The process of selecting our ensemble consists of a series of filtering steps, each of which produces a smaller sample of conformation space. Of course, any reduction in size can only result in a reduction of the quality with which some elements of the set can approximate the bound conformation. To evaluate our selection procedure, we performed a series of experiments to determine the quality of the sample of conformations at the end of each step, as well as to measure how this quality is affected by the reduction in size enforced by these steps.

### Quality of the rotamer representation of side-chains

Our first experiment addresses the following question: does limiting the set of conformations that we consider to those representable using our rotamer library, in and of itself prevent us from closely approximating the bound configuration? That is, given an unbound patch and our rotamer library, can we produce a configuration that is close to the bound conformation of that patch?

The fact that the bound and unbound backbone configurations might differ makes it difficult to answer this question directly. To remove the effect of the backbone difference, we graft side-chains in their bound conformation onto the unbound backbone (the *bound_UBB *conformation) of the patch and compare this with the most similar rotamers grafted onto the unbound backbone (the *rotamer_UBB *conformation), where similarity refers to proximity to the bound conformation. We produce the *bound_UBB *conformation as follows: for each patch residue we superimpose its bound state onto its unbound state using a best fit alignment of backbone atoms. Then we translate that residue's side-chain from the bound conformation so as to superimpose its C*_α _*atom onto the unbound C*_α_*. To produce the *rotamer UBB *conformation, we select from the rotamer library the closest rotamer to the bound conformation for each patch residue, as observed in *bound_UBB *(recall that as described in the Methods Section, a rotamer representing the unbound state is added to the rotamer sampling of any residue that has no rotamer from the library within 0.6 Å RMSD). We build these rotamers onto the unbound patch backbone. Note that these two constructs are used for analysis only, since they are derived using information from the bound state, which would not be available in a predictive setting.

**Figure 3:**
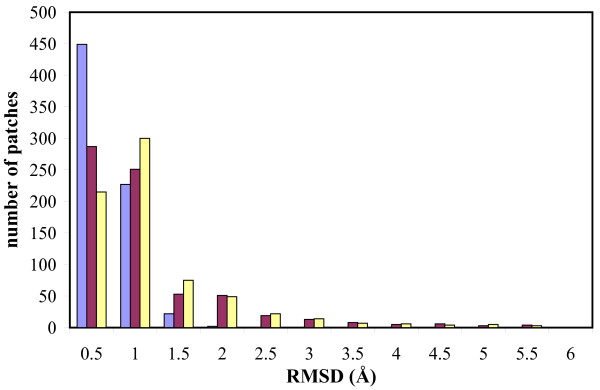
**Quality of our rotamer library**. We study the extent to which our set of rotamers can approximate the bound side-chains for our data set of 700 patches. For each patch we graft the bound side-chains onto the unbound backbone (*bound_UBB *conformation) and make an alternate graft of the closest rotamers in our rotamer library (*rotamer_UBB *conformation). The all-atom RMSDs between these grafts provide evidence that our rotamer library is extensive enough to produce a side-chain configuration near the bound. In all but 24 of the 700 patches this RMSD (blue, left bar) is within 1 Å. When we account for the changes in the backbone conformation between the bound and unbound conformations of the patch however, results are not as good. The maroon (center) and yellow (right) bars show the all-atom RMSD between the bound conformation of the patch and the *bound_UBB *and *rotamer_UBB *conformations, respectively. In 185 of the 700 patches, the *rotamer_UBB *conformation is more than 1 Å away from the bound conformation of the patch.

Figure 3 addresses the question of quality of the rotamer representation of side-chains by comparing all-atom RMSDs from our data set with and without backbone difference. We calculated the RMSD of the *bound_UBB *with respect to the *rotamer_UBB *for all 700 patches and found that for 614 of them the two conformations are within .7 Å of each other, for 676 patches they are within 1 Å, and none are found to be more than 1.6 Å from each other (see Figure 3). We conclude that the rotamer library is adequate for side-chain representation of the bound state, meaning that at least one configuration that can be built from the rotamer library is close enough to the bound conformation of the side-chains.

These results illustrate the adequacy of using a rotamer library to represent side-chain conformations, but they are somewhat misleading since they explicitly remove the effects of backbone flexibility. Therefore we also directly compute the all-atom RMSD between both the *bound_UBB *and the *rotamer_UBB *conformations and the bound conformation of the patch. We see in Figure 3 that the bound conformation of the patch is usually close to the *rotamer_UBB *conformation: for 515 of the 700 patches this RMSD is within 1 A. However, in 61 patches this RMSD is greater than 2 Å, twelve of these are above 4 Å with a high of 5.43 Å. We expect to to encounter difficulty in prediction when the patch backbone changes significantly. This is not unexpected when considering the results from the CAPRI experiment [32].

### Quality of the sample after SCMF filtering

**Figure 4:**
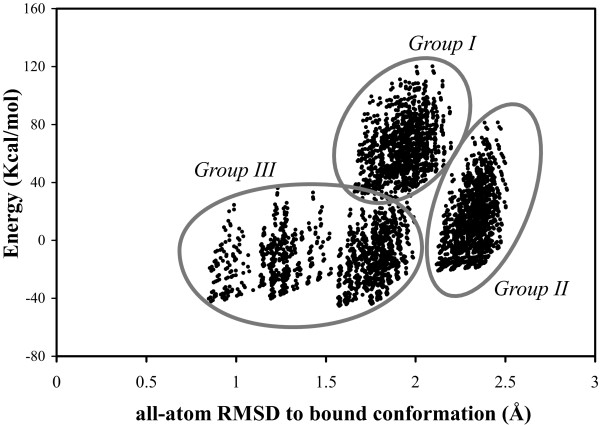
**Distribution of the SCMF-filtered conformations of a patch**. We consider a patch on the receptor of the complex 1AKJ (illustrated in Figure 1) and plot the energies of the corresponding 3888 filtered conformations versus their all-atom RMSDs to the bound conformation of the patch. We observe a clustering of these conformations in three main groups, labeled group I, group II, and group III that are mostly defined according to the values of the χ1 angle of one tryptophan in the patch, named T (see text for details). Note that this graph shows that many good conformations for the patch have been retained by the filtering (88 of these are within 1 Å RMSD of the bound conformation of the patch).

Having established that for 74% of our test cases at least one configuration based on our rotamer library is close to the bound conformation of the patch (97% if we ignore backbone flexibility), we next considered the set of conformations produced by the SCMF filtering step described in the section on SCMF filtering. To give a qualitative view of the results of this step, we first examine the filtered sample of conformations obtained for the typical patch on the receptor of the protein complex 1AKJ which appeared in Figure 1 and 2. This patch contains eight residues with flexible side-chains and one alanine whose side-chain is fixed, as we do not consider the hydrogen. In Figure 4 we plot the energies of the 3888 conformations of the patch that were selected by the SCMF filtering procedure as a function of their all-atom RMSDs to the bound conformation of the patch. Interestingly, the conformations in the filtered ensemble are not uniform but appear in distinct clusters in this Energy-RMSD space. Further analysis shows that these clusters are mostly determined by the χ_1 _angle of one tryptophan, named T, in the patch, which in the bound state has a χ_1 _angle of -59° and a χ_2 _angle of 114°. For all members of the cluster identified as Group I in Figure 4 T has a χ_1 _angle of 180° and a χ_2 _angle of -90°. This is the cluster whose elements have the highest energies ranging from 40 to 80 Kcal/mol. All members of group II are far from the bound conformation, with RMSDs higher than 2 Å; in all of these T has a χ_1 _angle of 60° and a χ_2 _angle of 90°. In group III, T has a χ_1 _of -60°, i.e. close to its value in the bound conformation. This set is the most dispersed with at least three apparent subgroups. Interestingly, those members of this group whose RMSD with the bound conformation is below 1.53 Å have a tryptophan T χ_2 _angle of 90°, while all those with RMSD above 1.53 Å have a χ_2 _angle of -90°. There are 88 patch conformations within 1 Å RMSD of the bound conformation. The behavior we describe here is not unique to 1AKJ: we observed the same clustering determined by the χ_1 _angle of an influential residue (usually the largest) for most of the 700 patches we studied.

In addition to the clustering of the different conformations of a patch, Figure 4 illustrates a good correlation between the energy of a conformation of a patch and its RMSD to the bound state, in the sense that the conformations with the lowest energies correspond to the conformations that most resemble the bound state. While this is a very desirable property (if it were to be consistently true it would mean that the conformation that is closest to the bound conformation could be easily identified), it was found unfortunately to be more qualitative than quantitative. When we checked all seven hundred patches in our test set, we found in fact that picking the conformations with the lowest energies did not guarantee that we would retain a conformation that resembles the bound state, unless we retained a very large number of conformations. This is by no means surprising: the energy function we use here is simplified and only include internal interactions, neglecting solvent and most importantly neglecting the effect of the partner protein in the complex, as the position of this partner is not known.

**Figure 5:**
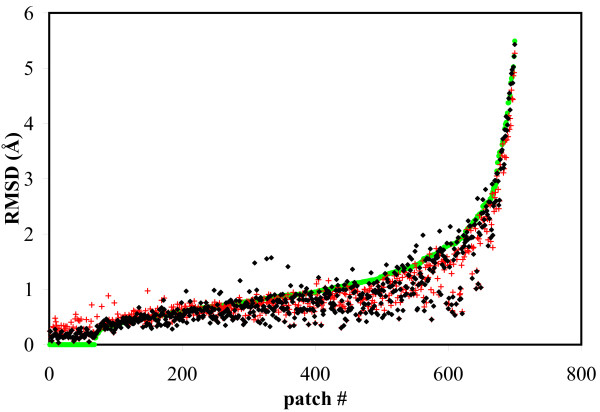
**Reducing sample size of test patches with SCMF**. All-atom RMSD between the unbound conformation (green dot), the best pick in the SCMF-filtered ensemble (red cross), the theoretical *rotamer_UBB *conformation (black diamond), and the bound state for all 700 patches in our data set. To improve clarity, these test cases are arranged along the x-axis in order of increasing RMSD between the unbound and bound conformations. For 521 of the 700 cases, the best pick is within 1 Å RMSD of the bound conformation. (Note that the unbound conformation is not yet added to ensemble). We observe that for some of the patches, the best pick is better than the *rotamer_UBB *conformation: this indicates that local arrangement of the side-chain does not always lead to a good conformation for the patch as a whole (see text for details).

Quantitatively, we can see that the SCMF filtering significantly reduces the size of the sample of conformations for any given patch while retaining many good docking candidates, i.e. conformations of the patch that are close (in RMSD) to its bound conformation. This is illustrated in Figure 5. Among the 700 test cases we consider, we found that for 521 of them, the filtered sample includes at least one conformation that is within 1 Å RMSD from the bound conformation, and for 644 of them, there is at least one conformation within 2 Å RMSD. (At a later stage we improve our results by adding the unbound conformation to the ensemble.) For 481 of our test cases, the closest conformation of the patch to the bound conformation in the filtered ensemble is better than the unbound conformation. This by itself validates our approach: it is possible to start with a better conformation than the unbound structure in docking experiments.

Figure 5 highlights another important aspect of our procedure: the best conformation generated for a patch is not always the one in which the conformation of each of its side-chains is as close as possible to its configuration in the bound state of the patch. Recall that for testing our rotamer library, we designed an artificial conformation of the patch, *rotamer_UBB*, in which each side-chain was represented by a rotamer that best matches the conformation observed in the bound state. We observe that in 476 cases, the best pick in the filtered ensemble is better than the *rotamer_UBB *conformation. In these cases, some of the side-chains in the best pick have shifted from what would be their best rotamer state according to *rotamer_UBB *to provide a better approximation for the patch as a whole.

There is a limit however to how much we can improve upon the unbound conformation (or even the theoretical *rotamer_UBB *conformation): when the backbone changes significantly between the unbound and bound states (i.e. with an all-atom RMSD larger than 2 Å), we could not find any good conformation in the patch ensembles.

### Quality of the sample after clustering

The size of the sample of conformations that passed the SCMF filter is still too large for this sample to be used in a docking experiment. This size varies from 27 to 331,776 in our test set of 700 patches, depending on the number and nature of the residues they include. We therefore used a final size-reducing step, *k*-means clustering, with *k *set to 50. Note that the behavior observed in Figure 4 suggests a clustering approach.

**Figure 6:**
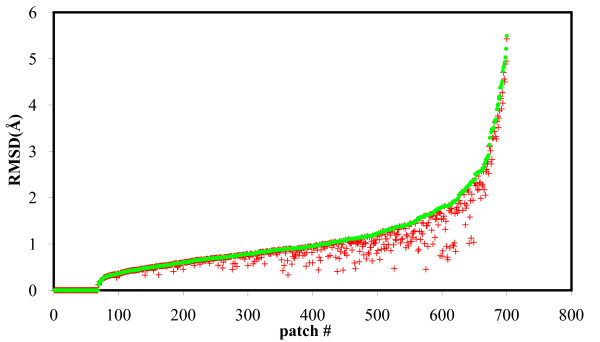
**Further reducing sample size of test patches with clustering**. All-atom RMSD between the unbound conformation (green dot), the best pick in the final ensemble after clustering (red cross), and the bound state for all 700 patches in our data set. We observe that in most cases, we are able to retain in our small sample of conformations for a patch a best pick that is better than the unbound conformation in representing the bound state.

Our final sample of conformations includes the representatives of the (up to) 50 clusters plus the unbound conformation of the patch. In Figure 6 we plot the all-atom RMSD between the best conformation in this final sample and the bound conformation of the corresponding patch, with the all-atom RMSD between the unbound and bound conformations serving as a reference. This figure presents our main result in this study. When the all-atom RMSD between the unbound and bound states is small (less than 0.7 Å), there is very little room for improvement on the unbound conformation. When the same RMSD however is between 0.7 and 2.4 Å, our sampling procedure usually finds a better configuration with smaller RMSD to the bound configuration. When the RMSD of unbound to bound is above 2.4 Å, our best representatives continue to do better than unbound, but this improvement is no longer significant: in these cases, there is a relatively large backbone displacement. Overall we see 480 cases, from the 700, in which the all-atom RMSD of the best representative in the sample to the bound conformation is less than 1 Å and 160 additional cases for which this RMSD is between 1 Å and 2 Å. In 650 of the 700 test patches we produce a candidate that is either within 1 Å RMSD of the bound conformation or is closer to the bound state than the unbound is. On average, we see an improvement of 0.12 Å for the best picks in our ensembles compared to the corresponding unbound conformations. The improvement increases as the backbone changes increase: we see an average RMSD improvement of 0.25 Å from unbound state for the cases where the backbone RMSD of unbound to bound is greater than 1 Å.

### Filtering does not damage the quality of our sample

**Figure 7:**
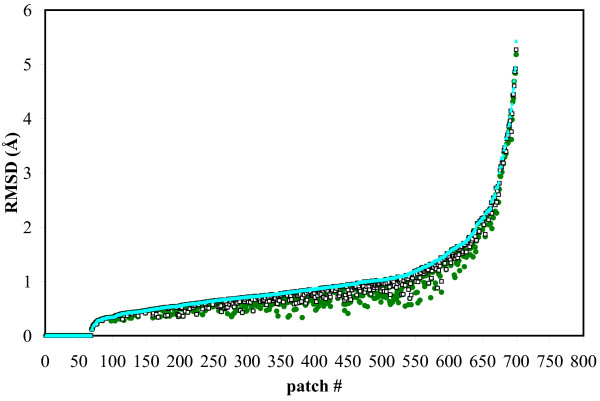
**Quantify loss of quality at each step in generating docking candidates**. All-atom RMSD between the best picks in the exhaustive ensemble (green dots), the SCMF-filtered ensemble (black boxes), the final ensemble after *k*-means clustering (cyan triangle), and the bound conformation of the corresponding patch. To improve clarity, the test cases are arranged along the x-axis in order of increasing RMSD between the best pick in the final ensemble and the bound conformation. For comparison purposes, the bound conformation has been added to both the exhaustive ensemble and to the SCMF-filtered ensemble, rather than only after clustering.

As mentioned earlier, we are well aware that any reduction in the size of the sample of conformations can only reduce the quality of the approximation provided by the best element of the sample. Figure 7 shows that we lose very little in approximation quality (as measured by all-atom RMSD) through both the SCMF filtering and the clustering stages. In the SCMF filtering, there is only one case in which nothing similar to the best pick in the exhaustive sample was retained; in this case, the best conformation after filtering is .59 Å RMSD away from the best possible pick when all degrees of freedom are considered. We noticed that this is due to a a 93° change in the χ_1 _angle of a tryptophan of the patch that was not energetically favorable unless the docking partner was taken into account. The largest loss in RMSD in the clustering step was .51 Å RMSD.

The failure to maintain a good conformation for the patch (i.e. similar to its bound conformation) in the filtered ensemble is therefore not a consequence of the filtering process: we observed that when the backbone of the patch changes less than 1 Å RMSD upon docking, we generate a conformation for the patch that is within 1 Å of its bound state in 78% of the 700 initial exhaustive ensembles, as compared with 75% of the SCMF filtered ensembles and 68% of the final ensembles. With backbone change in this range, 82% of the final ensembles are within 1.4 Å of the bound conformation. When the backbone changes are larger however, sampling side-chain configuration alone does not allow us to generate a conformation for the patch that resembles the bound state.

### Patch size and quality of the ensemble

As mentioned above, the number of effective residues included in a patch is limited to about 9, as a larger number would lead to combinatorial explosion that would become intractable at the clustering stage of our procedure. It is not clear, however, how this limit affects both our overall strategy for protein docking and the quality of the ensemble of conformations we generate for one patch.

We do not believe that patch size will significantly affect our ability to find good docking conformations, as we will generate patchworks of patches of the surfaces of the unbound proteins to ensure that we cover the interface region. This will be studied in more detail, however, as we develop the next steps of our procedure.

In addition, we do not believe that patch size has an effect on the quality of the ensemble of conformations we generate. We did test this assertion on the interface of the test case with PDB code 1B6C in our data set. We considered patches of varying sizes, from 4 to 15 effective residues covering the interface on the receptor protein. Ensembles were generated for each patch and filtered by SCMF. The quality of each ensemble was defined as the best RMSD to the bound state computed over all conformations contained in the ensemble. We found very little difference between the qualities of the patches for patches of different sizes covering the same interface. As for all 700 patches in our data set, we found that it is the extent to which the backbone changes between the unbound and bound structures of the patch that mainly defines the quality of the ensemble.

We also eliminated the possibility that edge effects might be important (i.e. the influence of the residues bordering the patch): we varied the weight of the energy contributions of these residues (whose conformations are fixed) from 0 (i.e no influence) to 1.0 (i.e. full contribution) and found no effects on the quality of all patches in the interface of 1B6C, for all sizes considered.

## Conclusion

This paper focuses on how to incorporate flexibility in protein-protein docking studies. We divide the surfaces of each protein into local patches and describe the effect of flexibility on each patch by sampling the space of conformations of its side-chains. Likely positions of individual side-chains are given by a rotamer library; this library is used to derive a sample of possible mutual conformations within the patch. We control the size of the sample using the so-called SCMF filtering that maintains broad coverage while selecting conformations with low energy, and by clustering similar configurations. We have shown that in most cases this procedure allows us to generate a good sampling of a patch conformation that includes at least one configuration that is close to the bound conformation of the patch. We have also shown that usually this best configuration is a better match to the bound state than the given unbound conformation of the patch.

Our procedure however breaks down for cases in which the backbone configuration for the residues in the patch changes significantly between the unbound and bound states. This points to a direction for future work. We expect an improvement in our sampling of the conformation of a patch if we account for backbone flexibility explicitly. Recently there has been progress in modeling backbone flexibility in the initial surface matching stage of docking. For example, in FlexDock [24,33], molecules are decomposed into rigid domains, which are docked separately and then the possible dockings are reconnected using a graph-based algorithm. RosettaDock [34,35] creates a "fold tree" representation of the molecular system which explicitly handles backbone flexibility. ATTRACT [36] adopts systematic protein-protein docking starting from conformations generated from normal mode analysis using a Gaussian network model to represent the protein. In future work we will investigate such methods to incorporate backbone flexibility into our ensembles where needed, again addressing the issue of computational complexity.

## Authors' contributions

All authors participated in the design of the study. PFL and SG performed the experiments. All authors helped to draft the manuscript. They all read and approved the final manuscript.
